# Gastrointestinal colonization with multidrug-resistant Gram-negative bacteria during extracorporeal membrane oxygenation: effect on the risk of subsequent infections and impact on patient outcome

**DOI:** 10.1186/s13613-019-0615-7

**Published:** 2019-12-18

**Authors:** Giacomo Grasselli, Vittorio Scaravilli, Laura Alagna, Michela Bombino, Stefano De Falco, Alessandra Bandera, Chiara Abbruzzese, Nicolò Patroniti, Andrea Gori, Antonio Pesenti

**Affiliations:** 1 Department of Anesthesia, Critical Care and Emergency, Fondazione IRCCS Ca’ Granda - Ospedale Maggiore Policlinico, Via F. Sforza 35, 20122 Milan, MI Italy; 2 Department of Pathophysiology and Transplantation, University of Milan, Milan, MI Italy; 3 Infectious Diseases Unit, IRCCS Ca’ Granda Ospedale Maggiore Policlinico Foundation, Milan, Italy; 4 Department of Anesthesia, Critical Care and Emergency, ASST Monza San Gerardo Hospital, Monza, MB Italy; 5 Anaesthesia and Intensive Care, San Martino Policlinico Hospital, IRCCS for Oncology, Genoa, Italy; 6 Department of Surgical Sciences and Integrated Diagnostics, University of Genoa, Genoa, Italy

**Keywords:** Retrospective study, Health care-associated infection, Extracorporeal membrane oxygenation, Multi-drug resistance, Colonization

## Abstract

**Background:**

In ICU patients, digestive tract colonization by multidrug-resistant (MDR) Gram-negative (G−) bacteria is a significant risk factor for the development of infections. In patients undergoing extracorporeal membrane oxygenation (ECMO), colonization by MDR bacteria and risk of subsequent nosocomial infections (NIs) have not been studied yet. The aim of this study is to evaluate the incidence, etiology, risk factors, impact on outcome of gastrointestinal colonization by MDR G− bacteria, and risk of subsequent infections in patients undergoing ECMO.

**Methods:**

This is a retrospective analysis of prospectively collected data: 105 consecutive patients, treated with ECMO, were admitted to the ICU of an Italian tertiary referral center (San Gerardo Hospital, Monza, Italy) from January 2010 to November 2015. Rectal swabs for MDR G− bacteria were cultured at admission and twice a week. Only colonization and NIs by MDR G− bacteria were analyzed.

**Results:**

Ninety-one included patients [48.5 (37–56) years old, 63% male, simplified acute physiology score II 37 (32–47)] underwent peripheral ECMO (87% veno-venous) for medical indications (79% ARDS). Nineteen (21%) patients were colonized by MDR G− bacteria. Male gender (OR 4.03, *p* = 0.029) and duration of mechanical ventilation (MV) before ECMO > 3 days (OR 3.57, *p* = 0.014) were associated with increased risk of colonization. Colonized patients had increased odds of infections by the colonizing germs (84% vs. 29%, *p* < 0.001, OR 12.9), longer ICU length of stay (LOS) (43 vs. 24 days, *p* = 0.002), MV (50 vs. 22 days, *p* < 0.001) and ECMO (28 vs. 12 days, *p* < 0.001), but did not have higher risk of death (survival rate 58% vs. 67%, *p* = 0.480, OR 0.68). Infected patients had almost halved ICU survival (46% vs. 78%, *p* < 0.001, OR 4.11).

**Conclusions:**

In patients undergoing ECMO for respiratory and/or circulatory failure, colonization by MDR G− bacteria is frequent and associated with more the tenfold odds for subsequent infection. Those infections are associated with an increased risk of death.

## Background

Extracorporeal membrane oxygenation (ECMO) is a life-support technique used in patients with potentially reversible refractory respiratory or circulatory failure [1]. Healthcare-associated infections (HAIs) are common in ECMO patients [2–4] due to several predisposing factors: intensive care unit (ICU) hospitalization, patients’ comorbidities, immunodeficiency induced by the critical illness and invasiveness of ECMO and other life-support procedures (e.g., invasive mechanical ventilation, renal replacement therapies). ECMO patients suffering from HAIs have longer ECMO runs, ICU length of stay (LOS), and higher mortality rate [3]. Recently, we reported that HAIs during ECMO are frequently caused by multidrug-resistant (MDR) Gram-negative (G−) bacteria [2], and we observed that patients infected by MDR bacteria had higher odds for death.

ICU patients have higher rates of digestive tract colonization by MDR G− bacteria (i.e., producing extended-spectrum β-lactamase (ESBL+) and carbapenem-resistant bacteria) compared to patients admitted to general wards [5, 6]. Such colonizations could represent a significant risk factor for the development of subsequent infections [7–9]. A growing body of evidence indicates that dysbiosis of the gut microbiota is common in critically ill patients and may play a crucial role in increasing the risk of gastrointestinal colonization. To our knowledge, the rate of colonization by MRD bacteria and the risk of subsequent infections have not been studied in ECMO patients. In such a fragile population, prevention, early diagnosis and prompt treatment of MDR HAIs may significantly affect morbidity and mortality.

The aim of the present study is to evaluate the incidence, risk factors, and impact on subsequent HAIs as well as clinical outcomes of digestive tract colonization by MDR G− bacteria in a large cohort of non-surgical patients undergoing ECMO for respiratory or circulatory failure.

## Methods

We present a retrospective analysis of prospectively collected data of all consecutive ECMO patients admitted to the General Intensive Care Unit (ICU) of San Gerardo Hospital (Monza, Italy) from January 2010 to November 2015. For further details on ECMO setting and patient care see Setting and Standard of Care, Additional file 1: Methods S1. Notably, at San Gerardo Hospital ICU, rectal swabs are collected and cultured for ESBL+ (i.e., *E. coli*, *Enterobacter* spp.) and carbapenem-resistant (i.e., *Acinetobacter* spp., *P. aeruginosa*, *K. pneumoniae* carbapenemase producing and other Enterobacteriaceae) G− bacteria at ICU admission and twice a week. We will refer to ESBL+ and carbapenem-resistant G− bacteria as “MDR G− bacteria”.

The Institutional Ethical Committee, and written informed consent was waived due to the retrospective observational design of the study. All patients receiving ECMO support were considered for inclusion. Exclusion criteria were: (1) ICU length of stay (LOS) < 24 h; (2) ECMO use < 24 h; (3) occurrence of a NI prior to ECMO connection; (4) missing medical records. At baseline, the following patients data and ECMO parameters were collected: demographics (i.e., gender, age); comorbidities [10]; immunocompromised status (i.e., chronic immunosuppressive therapies, active hematological malignancies, autoimmune diseases); diagnosis at admission; infections at admission; renal replacement therapy before ECMO cannulation; severity scores (i.e., Sequential Organ Failure Assessment—SOFA—score and Simplified Acute Physiology Score II—SAPS II of the first 24 h of ICU stay); PaO_2_/FiO_2_ at ECMO connection; ECMO configuration (i.e., veno-venous, veno-arterial, other); transfer from peripheral hospital; length of invasive mechanical ventilation (IMV) before ECMO connection; antimicrobial therapy (i.e., exposure to extended-spectrum penicillins with β-lactamase inhibitor or carbapenems).

The following outcomes were recorded: survival at ICU discharge, ICU LOS, duration of IMV, and duration of ECMO.

All the positive microbial cultures obtained from ICU admission until ICU dismissal have been independently evaluated in light of the available clinical, laboratory, and radiographic data by two specialized intensivists (VS and GG) and two infectious diseases specialists (AB and LA) following international guidelines [11–13]. The patients with rectal or perineal swabs positive for MDR G− bacteria were considered “colonized”. Similarly, patients with diagnostic criteria for ventilator-associated pneumonia (VAP), catheter-associated urinary tract infection (UTI), bloodstream infection (BSI), catheter-related bloodstream infection (CRBSI) (see Additional file 1: Table S1, Methods S1) [9] due to MDR G− bacteria were considered “infected”. Infections due to pathogens different from MDR G− bacteria were not considered in this analysis and have been described elsewhere [2].

### Statistical analysis

Due to the retrospective nature of the study, no statistical power calculation was performed a priori, and the sample size was equal to the number of patients treated during the recruitment period. Data are presented as median and interquartile range (IQR) for continuous variables. Categorical variables are expressed as number of patients (percentage of the subgroups). For binary outcome measures, odds ratios (OR) and associated 95% likelihood ratio-based confidence intervals were calculated, and the comparison between patients’ populations (i.e., colonized vs. non-colonized, infected vs. non-infected) were performed with Chi-square test or Fisher’s test, as appropriate. The Kruskal–Wallis test was utilized to compare non-parametric continuous variables between patients’ populations. Kaplan–Meier survival curve analysis was used with log-rank test for comparison of colonization-free and infection-free rates. Observations were right-censored.

Univariate regression models were applied to identify risk factors associated with colonization and infection. All subjects were included in the models, and follow-up began at the time of ECMO initiation. All statistical tests were 2-tailed, and statistical significance was accepted at *p* < 0.05. Analyses were performed using JMP 12.1 Pro (SAS, Cary, NC, USA) statistical program.

## Results

From January 2010 to November 2015, 105 patients were treated with ECMO at the General Intensive Care Unit (ICU) of San Gerardo Hospital (Monza, Italy). Ninety-one subjects (median age 49 years; 63% male) were included in the analysis. Reasons for exclusion of the remaining patients were: diagnosis of HAI prior to ECMO connection (10 patients), age < 18 years (2 subjects), ICU LOS and ECMO shorter than 24 h (2 patients) (see Additional file 1: Figure S1, Results).

Patients’ characteristics, comorbidities, and indications for ECMO support are summarized in Table 1, and Additional file 1: Table S2.

**Table 1:** Patients’ and treatment characteristics at the ECMO connection (*n* = 91)

Patients’ characteristics	Subgroups	Frequency or median
Age (years)		48.5 (37–56)
Gender (male)		58 (63%)
Weight (kg)		70 (65–85)
Charlson Comorbidity Index		1 (0–3)
Major comorbidities	Active smoker	26 (28%)
	Immunomodulating therapies^a^	22 (24%)
	Hematologic malignancies	13 (14%)
	COPD	10 (11%)
	Hepatopathy	10 (11%)
	Coronary artery disease	9 (10%)
	Diabetes	7 (8%)
	AIDS	3 (3%)
Transferred from peripheral hospital		76 (82%)
Transferred while on ECMO support		58 (63%)
Diagnosis and admission	ARDS	72 (78%)
	Cardiogenic shock	6 (7%)
	Asthma	4 (4%)
	COPD exacerbation	4 (4%)
	Septic shock	4 (4%)
	Other	2 (2%)
Infection at admission		65 (71%)
Autoimmune disease		8 (9%)
SAPS II		37 (32–47)
SOFA score		8 (6–11)
PaO_2_/FiO_2_ < 100 mmHg		70 (76%)
ECMO duration (days)		14 (8–27)
Veno-venous ECMO		80 (87%)
Low flow ECCO_2_R		8 (9%)
Femo-femoral cannulation		76 (83%)
ECMO circuits		2 (1–4)
IMV duration (days)		25 (12–44)
IMV duration prior to ECMO connection (days)		2 (1–6)
RRT during ECMO course		33 (36%)
RRT prior to ECMO connection		15 (16%)

Data are presented as absolute frequency (% of the included patients) or as median and interquartile range

*ECMO* extracorporeal membrane oxygenation, *COPD* chronic obstructive pulmonary disease, *ARDS* acute respiratory distress syndrome, *SAPS II* simplified acute physiology score, *SOFA* sequential organ failure assessment, *ECCO*_*2*_*R* extracorporeal carbon dioxide removal, *AIDS* acquired immunodeficiency syndrome, *IMV* invasive mechanical ventilation, *RRT* renal replacement therapy

^a^Including high-dosage corticosteroids, immunosuppressants or both

We analyzed a total of 1213 positive cultures. Nineteen (21%) patients were colonized by MDR G− bacteria. Of them, 11 (58%) were colonized by *A. baumannii*, 4 (21%) by *K. pneumoniae*, 2 (11%) by *P. aeruginosa*, and 2 (10%) by other Enterobacteriaceae. The clinical characteristics of colonized and non-colonized patients are depicted in Table 2. Factors associated with increased risk for colonization were male gender [OR 4.03 (1.08–15.0), *p* = 0.029] and duration of IMV before ECMO connection > 3 days [OR 3.57 (1.25–10.2), *p* = 0.014]. Among the other variables, ARDS and use of RRT prior to ECMO connection were associated with high, but not significant, OR estimates [i.e., 6.00 (0.74–48.2) and 1.48 (0.41–5.29), respectively]. Multivariable logistic regression was not deemed appropriate due to the small number of events (i.e., *n* = 19).

**Table 2:** Univariable logistic regression analyzing risk factor for colonization due to multidrug-resistant Gram-negative bacteria

Clinical characteristics	Colonized (*n* = 19)	Non-colonized (*n* = 72)	*p*	Odds ratio^a^ (95% CI)
Year				
2010	4 (21%)	9 (13%)	0.597	
2011	1 (5%)	13 (18%)		
2012	3 (16%)	10 (14%)		
2013	2 (11%)	13 (18%)		
2014	4 (21%)	15 (21%)		
2015	5 (26%)	12 (17%)		
Age (years)	52 (39–64)	46 (34–55)	0.075	1.03 (0.99–1.07)
Gender (male)	16 (84%)	41 (57%)	0.029	4.03 (1.08–15.0)
Charlson Comorbidity Index	2 (1–4)	1 (0–3)	0.408	1.08 (0.88–1.31)
Transferred from peripheral hospital	15 (79%)	60 (83%)	0.655	0.75 (0.21–2.65)
IMV > 3 days prior to ECMO connection	11 (58%)	20 (28%)	0.014	3.57 (1.25–10.2)
RRT prior to ECMO connection	4 (21%)	11 (15%)	0.546	1.48 (0.41–5.29)
SOFA score	8 (6–11)	8 (6–11)	0.657	0.97 (0.84–1.10)
SAPS II	39 (32–53)	36 (32–46)	0.469	1.01 (0.97–1.05)
ARDS	18 (95%)	54 (75%)	0.059	6.00 (0.74–48.2)
PaO_2_/FiO_2_ < 100 mmHg	16 (84%)	54 (75%)	0.397	1.77 (0.46–6.81)
Infection at admission	15 (79%)	49 (68%)	0.355	1.76 (0.52–5.89)
Chronic immunosuppression^b^	4 (21%)	16 (22%)	0.912	0.93 (0.27–3.20)
Veno-venous support	16 (84%)	63 (88%)	0.706	0.76 (0.18–3.14)
Exposure to piperacillin/tazobactam	8 (42%)	40 (56%)	0.296	0.58 (0.21–1.62)
Exposure to carbapenems	10 (53%)	39 (54%)	0.905	0.94 (0.34–2.58)

Data are presented as absolute frequency (% of the included patients) or as median and interquartile range

*IMV* invasive mechanical ventilation, *ECMO* extracorporeal membrane oxygenation, *RRT* renal replacement therapy, *SOFA* sequential organ failure assessment, *SAPS II* simplified acute physiology score, *ARDS* acute respiratory distress syndrome

^a^Odds ratio of continuous variables are odds ratio per unit increase in variable

^b^Including high-dosage corticosteroids, immunosuppressants or both. Statistically significant results are highlighted in italic

**Fig. 1:**
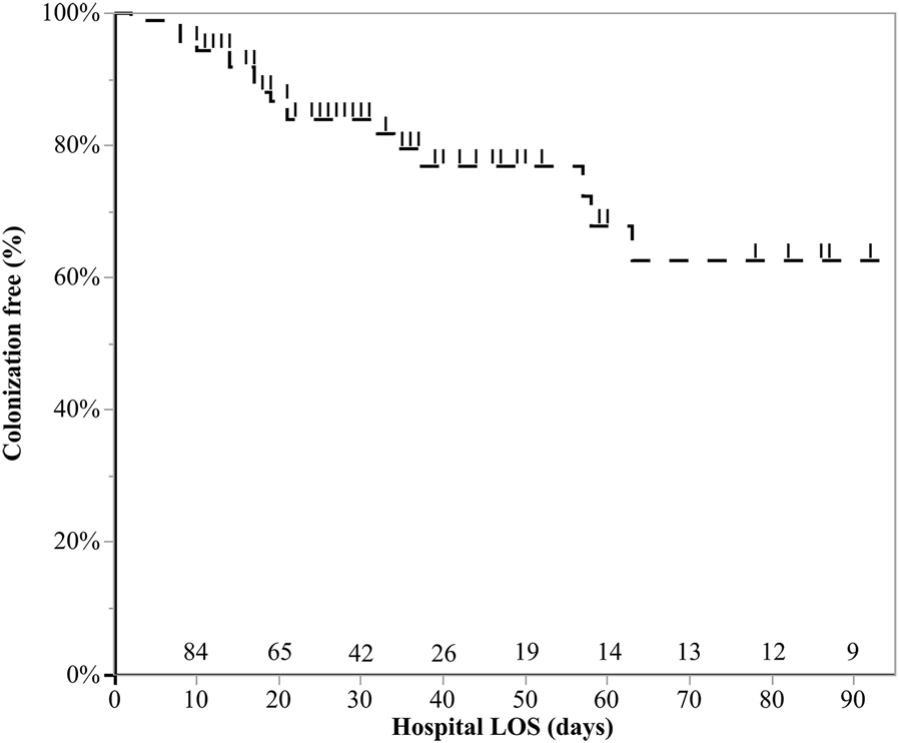
Probability of being colonization-free. Kaplan–Meier estimates of the unadjusted cumulative probability of being colonization-free (tracked line). Tick marks represent censored patients

Colonization was diagnosed after a median interval of 13 (1–22), 16 (8–32) and 17 (10–34) days from ECMO connection, institution of IMV, and hospital admission, respectively. Of note, 5 (26%) of the colonization were diagnosed in the first three days after ICU admission. The time course of colonization is depicted in Fig. 1.

Colonized patients had significantly longer ICU LOS [i.e., 43 (23–84) vs. 24 (12–37), *p* = 0.002], longer IMV [i.e., 50 (23–89) vs. 22 (10–37) days, *p* < 0.001] and longer ECMO support [i.e., 28 (16–60) vs. 12 (6–24), *p* < 0.001]. Colonization also increased the risk of need for tracheostomy [i.e., 68% vs. 28%, *p* < 0.001, OR 5.63 (1.88–16.8)]. In our cohort colonized patients did not have a higher risk of death than not colonized patients [survival rate 58% vs. 67%, *p* = 0.480, OR 0.68 (0.24–1.93)], but they had more than tenfold odds of developing an infection caused by the colonizing germs [84% vs. 29%, *p* < 0.001, OR 12.9 (3.41–49.1)] with high sensitivity [0.432 (95% CI 0.287–0.591)] and specificity [0.944 (95% CI 0.849–0.981)] of prior colonization to predict subsequent MDR G− bacterial infection.

In colonized patients, we observed 13 VAP, 2 UTI, and 1 CRBSI at a median of 11 (4–22) days from the day of diagnosis of colonization and 24 (13–60), 33 (19–47) and 34 (20–64) days from ECMO connection, intubation and hospital admission, respectively.

Overall, 37/91 patients (41%) developed an infection due to MDR G− bacteria (see Additional file 1: Figure S2, Tables S3, S4), which occurred at a median of 16 (7–31), 22 (12–45) and 25 (15–40) days from ECMO connection, intubation and hospital admission. The most common infection was VAP due to *A. baumannii*, both in the colonized (*n* = 7/16 infected patients, 44%) and non-colonized (*n* = 5/21 infected patients, 25%).

**Fig. 2:**
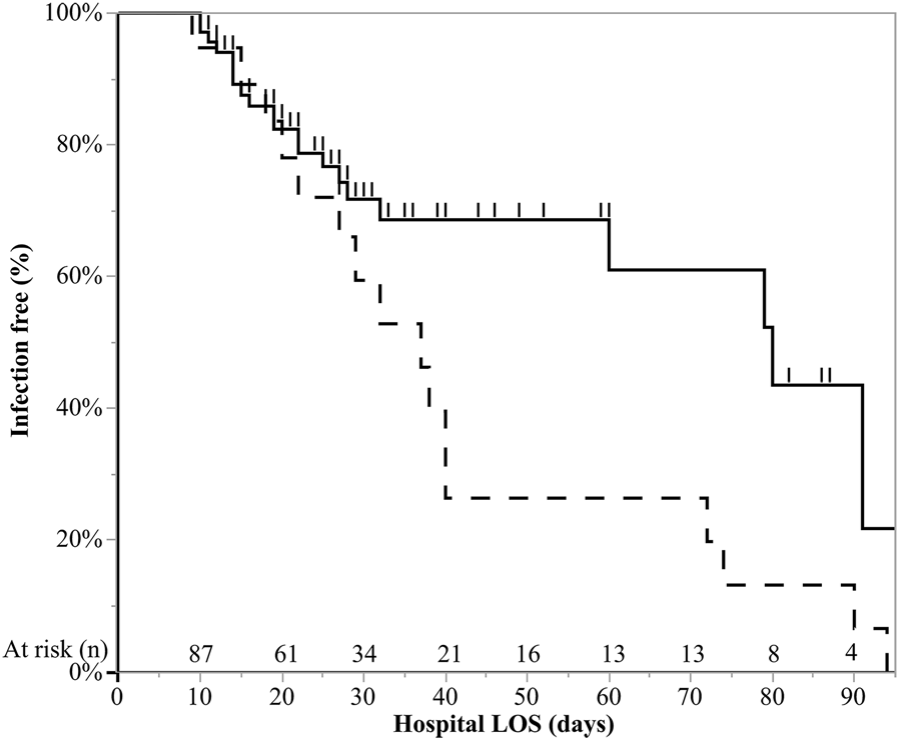
Probability of being infection-free. Kaplan–Meier estimates of the unadjusted cumulative probability of being infection-free. Tracked line represent colonized patients, tick line represent non-colonized patients. Tick marks represent censored patients

The clinical characteristics of infected and non-infected patients are depicted in Table 3. Factors associated with increased risk for infection were male gender [OR 2.68 (1.09–7.00), *p* = 0.030], duration of IMV before ECMO connection > 3 days [OR 7.33 (2.86–20.3), *p* = 0.001], use of RRT prior to ECMO connection [OR 5.29 (1.63–20.6), *p* = 0.001], diagnosis of ARDS [OR 8.04 (2.09–53.0), *p* = 0.001], infection at admission [OR 3.28 (1.22–9.93), *p* = 0.017] and colonization [OR 12.9 (3.83–59.9, *p* = 0.001]. Kaplan–Meier survival curve analysis with log-rank test showed a significant difference in the time course of infection between colonized and non-colonized patients (*p* = 0.025), indicating that infections developed earlier in colonized patients (Fig. 2).

**Table 3:** Univariable logistic regression analyzing risk factor for infection due to multidrug-resistant Gram-negative bacteria

Clinical characteristics	Infected (*n* = 37)	Non-infected (*n* = 54)	*p*	Odds ratio^a^ (95% CI)
Year				
2010	5 (14%)	8 (15%)	0.970	
2011	6 (16%)	8 (15%)		
2012	4 (11%)	9 (17%)		
2013	6 (16%)	9 (17%)		
2014	8 (22%)	11 (20%)		
2015	8 (22%)	9 (17%)		
Age (years)	50 (37–55)	47 (36–58)	0.587	1.00 (0.98–1.03)
Gender (male)	28 (76%)	29 (54%)	0.030	2.68 (1.09–7.00)
Charlson Comorbidity Index	1 (0–3)	1 (0–3)	0.839	1.21 (0.17–7.82)
Transferred from peripheral hospital	32 (86%)	43 (80%)	0.393	1.63 (0.53–5.62)
IMV > 3 days prior to ECMO connection	22 (59%)	9 (17%)	0.001	7.33 (2.86–20.3)
RRT prior to ECMO connection	11 (30%)	4 (7%)	0.005	5.29 (1.63–20.6)
SOFA	8 (6–12)	8 (6–11)	0.348	1.05 (0.94–1.17)
SAPS II	38 (32–49)	37 (30–43)	0.806	1.00 (0.97–1.03)
ARDS	35 (95%)	37 (69%)	0.001	8.04 (2.09–53.0)
PaO_2_/FiO_2_ < 100 mmHg	32 (86%)	38 (70%)	0.066	2.69 (0.93–8.97)
Infection at admission	31 (84%)	33 (61%)	0.017	3.28 (1.22–9.93)
Chronic immunosuppression^b^	8 (78%)	12 (78%)	0.946	0.96 (0.34–2.63)
Veno-venous support	34 (92%)	45 (83%)	0.223	2.26 (0.62–10.8)
Colonization	16 (43%)	3 (6%)	0.001	12.9 (3.83–59.9)

Data are presented as absolute frequency (% of the included patients) or as median and interquartile range

*IMV* invasive mechanical ventilation, *ECMO* extracorporeal membrane oxygenation, *RRT* renal replacement therapy, *SOFA* sequential organ failure assessment, *SAPS II* simplified acute physiology score, *ARDS* acute respiratory distress syndrome, *ICU* intensive care unit, *LOS* length of stay

^a^Odds ratio of continuous variables are odds ratio per unit increase in variable

^b^Including high-dosage corticosteroids, immunosuppressants or both. Statistically significant results are highlighted in italic

Infected patients had longer ECMO support [i.e., 27 (14–61) vs. 10 (5–16), *p* < 0.001], longer IMV [i.e., 44 (25–87) vs. 18 (9–31), *p* < 0.001] and longer ICU LOS [i.e., 43 (25–83) vs. 19 (10–29), *p* < 0.001]. Moreover, infected patients had increased needs for RRT [i.e., 22 (59%) vs. 11 (20%), *p* < 0.001, OR 5.73 (2.31–15.6)] and tracheostomy [i.e., 18 (49%) vs. 15 (28%), *p* = 0.042, OR 2.46 (1.03–6.01)]. Finally, infected patients had almost halved ICU survival [i.e., 17 (46%) vs. 42 (78%), *p* < 0.001, OR 4.11 (1.68–10.5)].

The time course of antibiotic use is shown in Additional file 1: Table S5. β-Lactam/β-lactamase inhibitors and antipseudomonal carbapenems were the most commonly employed antibiotic classes at time of perineal and rectal swab collection, while antipseudomonal carbapenems and colistin were the most common classes of antibiotics introduced as empiric and cultured-targeted therapy.

## Discussion

In the present study, we analyzed the epidemiology of digestive tract colonization by MDR G− bacteria in a large cohort of non-surgical patients undergoing ECMO for respiratory or circulatory failure. MDR G− bacteria colonization was highly frequent (21% of the patients), and risk factors associated with colonization were male sex and the need for prolonged (i.e., > 3 days) IMV prior to ECMO connection. Colonized patients had more than tenfold odds for subsequent infection by MDR G− bacteria, and those infections (mainly VAP due to *A. baumannii*) were associated with an increased risk of death [14].

In a previous analysis [2], we observed that up to 55% of ECMO patients develop at least an infectious complication, which is associated with worse clinical outcomes. Of those infections, 56% were caused by MDR bacteria, particularly non-fermenting G− bacteria. At San Gerardo Hospital general ICU, rectal swabs are routinely collected and cultured for MDR G− bacteria at ICU admission and twice a week. We hypothesized that, in patients undergoing ECMO, digestive tract colonization might precede infection and thus we retrospectively evaluated such cultures and their relationship with subsequent infections. To our knowledge, this is the first study evaluating the relationship between multidrug resistant bacteria gut colonization and infections in a relatively large cohort of ECMO patients.

Recent studies performed in European ICUs [15, 16], described colonization by MDR G− pathogens to occur in 2–10% of the patients. The higher rate of colonization documented in our patient population may be explained by several factors. First, in the last decade, Mediterranean countries and Italy, in particular, have been plagued by an epidemic of MDR bacteria in hospitalized patients and even in the general population [17, 18], with ESBL+ colonization reaching up to 50% of the patients [19]. Second, the high rate of colonization may reflect the invasiveness of treatment [20, 21] of our patients. Indeed, all our patients were invasively ventilated, and up to 50% underwent CRRT. We observed that longer duration of IMV prior to ECMO and male gender were associated with increased risk of colonization, while the use of RRT before ECMO cannulation had elevated though non-significant odds ratios for colonization. Third, ECMO patients usually receive broad-spectrum antimicrobials, which increase the risk of selection of MDR germs [9, 22]. Contrary to previous literature [23, 24], in our patients’ cohort exposure to carbapenems and extended-spectrum β-lactams/β-lactamase inhibitors and severity of illness (SOFA, SAPS II and PaO_2_/FiO_2_ ratio) were not associated with an increased risk of colonization by G− MDR bacteria. Finally, both invasiveness of care [21] and critical illness itself [25] alter the patients’ microbiota (i.e., lower diversity, lower abundance of commensals genera, overgrowth by single genera), limiting the protective role of microbiome thus increasing susceptibility to infection [26].

As previously documented [8, 9, 27], colonization by MDR G− bacteria was independently associated with increased odds for subsequent infection from the colonizing bacteria. While colonization per se was not associated with an increased risk of death, colonized patients had increased length of mechanical ventilation and ICU stay. In addition, infected patients had more than fourfold odds of death as compared to non-infected patients. In our opinion, the finding that infection but not colonization is associated with an increased risk of death is of utmost clinical interest. Treatment of bacterial colonization with broad-spectrum antibiotics is not recommended in patients with critical illness-associated immune dysfunction since it usually does not achieve the eradication of colonizing germs, while it instead increases evolutionary pressure towards the selection of multidrug resistant bacteria [28, 29]. Contrarily, management of colonization should aim at (1) early recognizing colonization through active screening protocols and molecular biology techniques [30]; (2) limiting the spread of MDR bacteria (by enforcing hand hygiene, contact precautions and cohort isolation [31]), and ideally, (3) avoiding development of infection in colonized patients; (4) institute patient-specific antibiotic therapy when a new infection would develop [28]. To reach the latest goal, immunologic profiling [32] of patients at highest risk for progression from colonization to infection would be crucial, also to guide the eventual institution of immunomodulating treatments. Also, we believe that gut microbiota may be a relevant therapeutic target for specific interventions (such as probiotics administration, decolonization strategies, etc.) that might contribute to reducing the risk of digestive tract colonization by MDR bacteria. Further prospective observational studies are needed to evaluate such aspects.

In our patient cohort, colonization by MDR G− bacteria occurred in patients with longer ICU stay, longer IMV and higher invasiveness of care, but was not associated with an increased risk of death. As such, colonization may be considered as a proxy for a more complicated clinical course, rather than a causative determinant of the unfavorable clinical course.

The main limitation of our study is its retrospective and single-center nature, which precludes the generalization of the results to the overall population of medical ECMO patients. Moreover, colonization due to MDR G+ bacteria (namely methicillin-resistant *Staphylococcus aureus*) was not evaluated since routine surveillance for MRSA colonization is not performed at San Gerardo Hospital General ICU due to the limited incidence of such infection. Finally, since around 25% of the colonizations occurred in the first 3 days from ICU admission, in this specific subgroup, we cannot clearly distinguish between community-acquired and hospital-acquired colonization, as most of our patients were admitted from peripheral hospitals, where surveillance was rarely performed.

## Conclusions

In a large cohort of non-surgical patients undergoing ECMO for respiratory and/or circulatory failure, colonization by MDR G− bacteria was frequent, associated with male sex and with prolonged duration (i.e., > 3 days) of IMV prior to ECMO connection. Colonized patients had more than tenfold odds for subsequent infection by MDR G− bacteria, and those infections were associated with an increased risk of death.

## Supplementary information

**Additional file 1: Methods S1.** Setting and standard of care. **Table S1.** Diagnostic criteria for infections. **Results**—**Figure S1.** Patients population flowchart. **Table S2.** Infections at admission. **Table S3.** Cox regression of the independent risk factors associated to the first NI. **Table S4.** Infection onset times.

## Data Availability

All the anonymized raw database is available after request to the corresponding author.
