# Supplementary material for: Gastrointestinal colonization with multidrug-resistant Gram-negative bacteria during extracorporeal membrane oxygenation: effect on the risk of subsequent infections and impact on patient outcome
Source: Ann Intensive Care. 2019 Dec 18;9:141. doi: 10.1186/s13613-019-0615-7 (PMC6920277; doi:10.1186/s13613-019-0615-7)

**Additional Material**

**Additional Methods**

*Setting and Standard of Care*

The General ICU of San Gerardo Hospital (Monza, Italy) is a 30-years ECMO experienced 8-10 beds (double-bed rooms) medical-surgical referral center for the treatment of respiratory failure [1] with roughly 550 patients per year.

Standard of care for ECMO patients at General ICU of San Gerardo Hospital comprises: orotracheal intubation and eventual late tracheostomy; nasotracheal feeding tube; urinary indwelling catheter; arterial, central venous and pulmonary artery catheterization. The detailed description of the management of ECMO support carried out at San Gerardo Hospital General ICU has been described elsewhere [2, 3](ref 20872350, 22183518). Notably, patients are always managed by peripheral percutaneous cannulation and connected to circuits featuring heparin-coated tubing, centrifugal pump, and polymethylpentene hollow fiber membrane oxygenators.

The following briefly describes the approach for infection control and prevention at General ICU of San Gerardo Hospital. No prophylactic antibiotic is provided at ECMO cannulation. Ventilator-associated pneumonia (VAP) prevention bundles are applied [4]: 1) semi-recumbent position; 2) chlorhexidine (0.15%) oral decontamination 3 times a day; 3) systematic stress ulcer prophylaxis; 4) systematic deep vein thrombosis prophylaxis. No prophylactic or selective decontamination antibiotic regimens are employed. Early enteral feeding is pursued. Catheters and cannulas insertion sites are monitored daily. Transparent dressings are applied routinely. Infusion and monitoring lines are replaced every 96 hours. Needle-free closed systems for drug infusion and blood withdrawal are utilized. No scheduled indwelling catheters removal is applied.

Infection control measures are applied [5]. Alcohol based hand hygiene is implemented. Whole body chlorhexidine bathing is applied at admission and every day after that [6]. Strict individual contact precautions and patients cohort isolation are applied to all patients since admission until colonization and/or infection by multidrug resistant bacteria is ruled out [7]. Staff cohorting is implemented [8]. Surveillance perineal swabs are cultured for A. baumannii and multidrug resistant (MDR) K. pneumoniae at admission and weekly thereafter. The occurrence of any MDR infection or colonization leads to restoring of the aforementioned isolation measures.

Besides surveillance perineal swabs, no routine tracheal, blood, urine cultures are performed. Management of septic patients follows internationally accepted guidelines [9]. No antibiotic prescription protocol is enforced, but Infection Disease Society of America (IDSA) Guidelines [10–12] for diagnosis and treatment of nosocomial infections (NIs) are consistently followed. Moreover, antibiotic regimens are: 1) monitored through an institutional antimicrobial stewardship implying written approval by the pharmacy service for the prescription of selected agents; 2) revised daily with a dedicated infectious diseases specialist consultant subsequent to a daily communication with the microbiology laboratory.

Table S1. Diagnostic criteria for infections.

| **Infection** | **Site of Culture** | **Bacterial Load** | **Clinical Signs** | **Also** |
| --- | --- | --- | --- | --- |
| **Blood Stream Infection** | 2 percutaneous blood samples  +  eventual blood from catheters | --- | Fever/tachycardia/hypotension  +  No further sign of localized infection | No differential time to positivity between percutaneous and catheters |
| **Catheter-related Blood Stream Infection^1^** | 2 percutaneous  +  catheter blood  or  catheter tip | --- | Fever/tachycardia/hypotension  +  No further sign of localized infection. Eventual erythema, swelling, purulent drainage from catheter insertion-site. | Differential time to positivity > 2 hours  or  catheter CFU > 3-fold percutaneous CFU  or  positive catheter tip |
| **Ventilator-Associated Pneumonia^2^** | Bronchoalveolar lavage | ≥ 10^4^ CFU/mL | 2 of: fever, leukocytosis/leucopenia, purulent secretions  +  New/progressive radiographic infiltrate  +  Worsening oxygenation | |
|  | Endotracheal Aspirate | ≥ 10^6^ CFU/mL |  |  |
| **Invasive Pulmonary Aspergillosis** | Proven: 1) microscopic analysis of needle aspiration/biopsy showing histopatologic/cytopatologic/direct microscopic evidence of hyphae and tissue damage or 2) positive culture of lung biopsy for Aspergillus spp. | | | |
|  | Putative: 1) bronchoalveolar lavage positive for Aspergillus spp. (with branching hyphae) without bacterial growth; 2) one of: refractory/recrudescent fever despite appropriate antibiotic therapy, pleuritic chest pain, pleuritic rub, dyspnea, hemophtysis, worsening oxygenation; 3) abnormal imaging by portable chest X-ray or computed tomography; 4) host risk factors (i.e. neutropenia, hematological malignancy, glucocorticoid treatment, congenital/acquired immunodeficiency). | | | |
| **Urinary Tract Infection^3^** | 2 consecutive urine specimens^4^ | ≥ 10^5^ CFU/mL | Fever/tachycardia/hypotension | |
| **Clostridium Difficile Colitis** | Unformed stool culture | --- | Fever/tachycardia/hypotension  +  Unformed stool | Enzyme immunoassay positive for C. difficile toxin A and B |

Colony forming units, CFU; ^1^) At least 48 hours after catheter positioning. ^2^) At least 48 hours after intubation. ^3^) At least 48 hours after catheterization. All the patients had urinary indwelling catheters. ^4^) Urinary catheter is removed after finding of a positive specimen, a new catheter is repositioned and a second specimen is collected.

**Additional Results**

**Figure S1.**

**
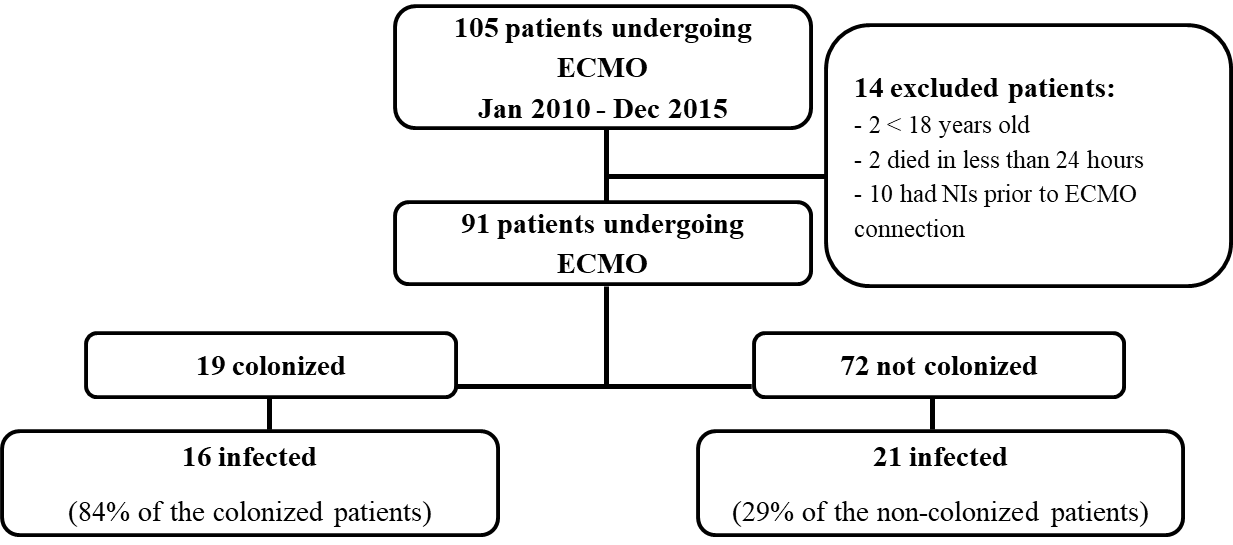
**

**Figure S1. Patients population flowchart.** ARDS, acute respiratory distress syndrome; ECMO, extracorporeal membrane oxygenation.

**Table S2. Infections at admission**

|  |  |  | **Overall**  **n = 65** |
| --- | --- | --- | --- |
| **Community Acquired Pneumonia** | Viral | Influenza A (H1N1) | 17 (26.5%) |
|  |  | Metapneumovirus | 2 (3.0%) |
|  |  | Adenovirus | 1 (1.5%) |
|  |  | Cytomegalovirus | 1 (1.5%) |
|  |  | Respiratory Syncytial Virus | 1 (1.5%) |
|  |  | Varicella Zoster Virus | 1 (1.5%) |
|  | Bacterial | S. pneumoniae | 5 (7.6%) |
|  |  | L. pneumophila | 4 (6.1%) |
|  |  | P. aeruginosa | 4 (6.1%) |
|  |  | M. pneumoniae | 3 (4.6%) |
|  |  | Meticillin Sensible S. aureus | 3 (4.6%) |
|  |  | S. aureus PVL+ | 1 (1.5%) |
|  |  | R. mucilaginosa | 1 (1.5%) |
|  |  | S. haemoliticus | 1 (1.5%) |
|  |  | A. baumannii | 1 (1.5%) |
|  | Unknown |  | 7 (10.7%) |
|  | Overall |  | 53 (81.4%) |
| **Septic Shock** |  | S. pneumoniae | 1 (1.4%) |
|  |  | S. aureus | 1 (1.4%) |
|  |  | S. pyogenes | 1 (1.4%) |
|  | Overall |  | 3 (4.6%) |
| **Endocarditis** |  | Echovirus | 1 (1.4%) |
| **Urinary tract infectiona** |  | E. coli | 3 (4.6%) |
| **Other** |  | M. tubercolosis | 3 (4.6%) |
|  |  | P. jiroveci | 1 (1.5%) |

PVL+, Panton-Valantine leukocidin.

**Table S3. Etiology of infections in colonized and non-colonized patients’ cohorts.**

|  |  | **Patients’ cohort** | |
| --- | --- | --- | --- |
| **Infection type** | **Agent** | **Non-Colonized** | **Colonized** |
| **VAP** | **A. baumannii** | 5 (25) | 7 (44) |
|  | **Pseudomonas spp.** | 4 (20) | 1 (6) |
|  | **Enterobacter** | 4 (20) | 2 (12) |
|  | **K. pneumniae** | 1 (5) | 3 (19) |
| **BSI** | **A. baumannii** | 1 (5) | 0 (0) |
|  | **Pseudomonas spp.** | 2 (5) | 0 (0) |
| **IVU** | **A. baumannii** | 1 (5) | 1 (6) |
|  | **Pseudomonas spp.** | 2 (2) | 1 (6) |
|  | **Enterobacter** | 1 (5) | 0 (0) |
| **Site infection** | **Pseudomonas spp** | 1 (5) | 0 (0) |
|  | **K. pneumoniae** | 1 (5) | 0 (0) |
| **CRBSI** | **K. pneumoniae** | 0 (0) | 1 (6) |

Data are presented as absolute frequency (% of the subgroup). VAP, Ventilator-Associated Pneumonia; UTI, Urinary Tract Infection; BSI, Blood Stream Infection; CRBSI, Catheter-Related Blood Stream Infection.

**Table S4**. Infection onset times.

|  | **VAP** | **UTI** | **BSI** | **CRBSI** | **P** |
| --- | --- | --- | --- | --- | --- |
| **Days from Hospital Admission** | 17 (10 - 30) | 18 (11 - 29) | 16 (9 - 24) | 16 (15 - 29) | 0.929 |
| **Days from Intubation** | 13 (7 - 26) | 16 (7 - 26) | 9 (6 - 13) | 14 (13 - 29) | 0.591 |
| **Days from ECMO connection** | 9 (3 - 18) | 9 (4 - 20) | 7 (2 - 9) | 10 (1 - 22) | 0.649 |

Data are presented as median and interquartile range. VAP, Ventilator-Associated Pneumonia; UTI, Urinary Tract Infection; BSI, Blood Stream Infection; CRBSI, Catheter-Related Blood Stream Infection; ECMO, extracorporeal membrane oxygenation.

**Table S5**. Use of antibiotics for the first nosocomial infection.

|  | **Ongoing at time of sample collection** | **Introduced as empiric therapy** | **Introduced after cultures results** |
| --- | --- | --- | --- |
|  | **No. (%) of infected patients** | | |
| **Overall use of antibiotics** | 49 (94%) | 24 (46%) | 30 (58%) |
| **Antibiotic Class** |  |  |  |
| **β-lactam/ β-lactamase inhibitors** | 15 (29%) | 2 (4%) | 6 (12%) |
| **Antipseudomonal carbapenems** | 12 (23%) | 9 (17%) | 11 (21%) |
| **Antivirals** | 11 (21%) | 1 (2%) | 0 (0%) |
| **Antifungals** | 10 (19%) | 2 (4%) | 6 (12%) |
| **Linezolid** | 8 (15%) | 5 (10%) | 3 (6%) |
| **Antipseudomonal fluoroquinolones** | 7 (13%) | 2 (4%) | 2 (4%) |
| **Antipseudomonal cephalosporins** | 5 (10%) | 4 (8%) | 3 (6%) |
| **Trimethoprim/sulfamethoxazole** | 5 (10%) | 1 (2%) | 1 (2%) |
| **Glycopeptides** | 4 (8%) | 2 (4%) | 3 (6%) |
| **Colistin** | 3 (6%) | 7 (13%) | 8 (15%) |
| **Macrolides** | 3 (6%) | 0 (0%) | 0 (0%) |
| **Tigecyclin** | 2 (4%) | 5 (10%) | 6 (12%) |
| **Aminoglycosides** | 2 (4%) | 1 (2%) | 1 (2%) |
| **Others^1^** | 11 (21%) | 4 (8%) | 5 (10%) |

^1^) Including 1^st^ and 2^nd^ generation cephalosporins, metronidazole, anti-staphylococcal penicillins, anti-tuberculosis agents, lincosamides, fosfomycin, rifampicin.

**Figure S2. Kernel density plot (violin plot) for infections onset time.** Black tick marks represent median onset time. *) *p*< 0.05 versus Gram- infections.


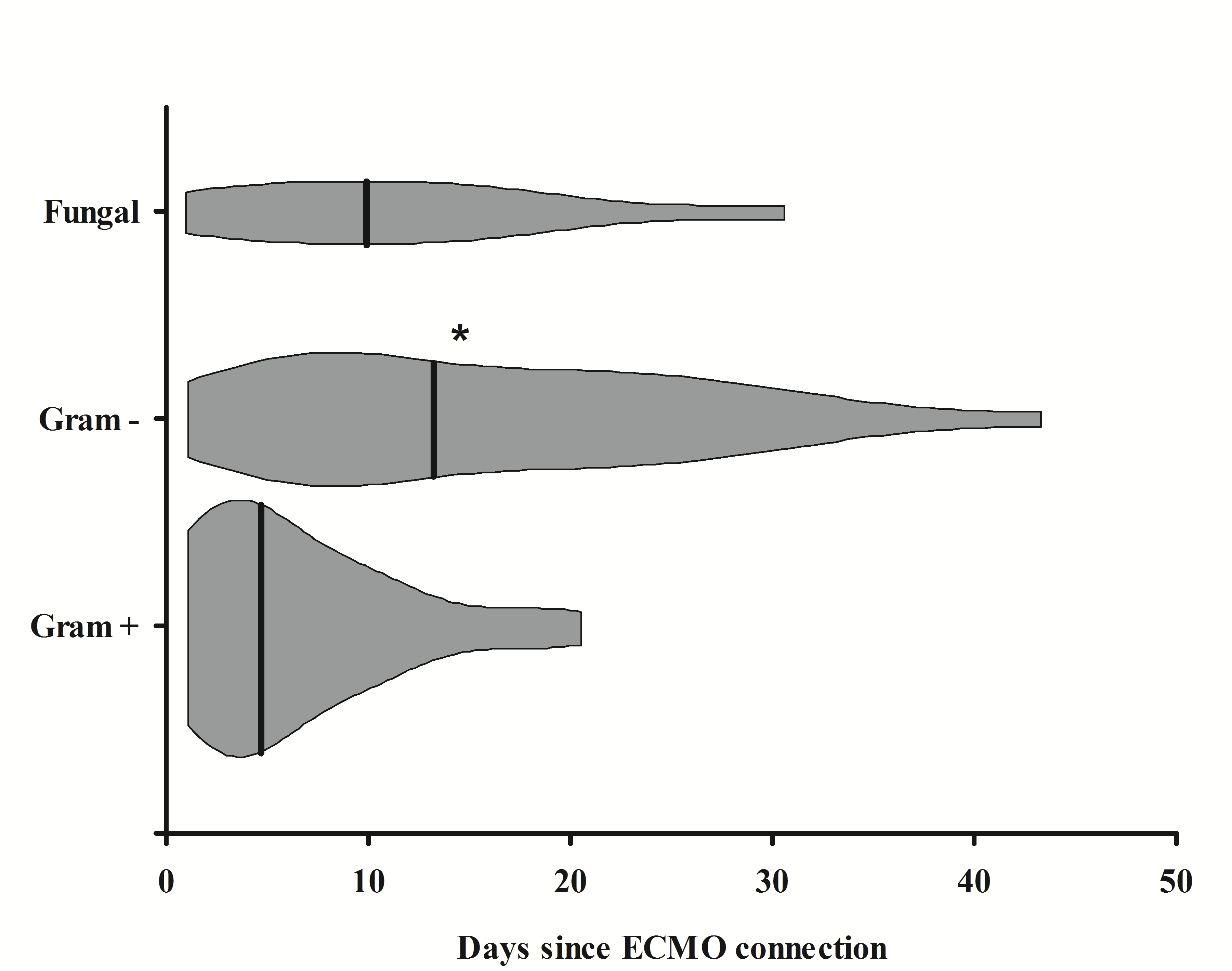

Supplement: Supplementary file 1 — Additional file 1: Methods S1. Setting and standard of care. Table S1. Diagnostic criteria for infections. Results—Figure S1. Patients population flowchart. Table S2. Infections at admission. Table S3. Cox regression of the independent risk factors associated to the first NI. Table S4. Infection onset times. [file 13613_2019_615_MOESM1_ESM.doc]
